# Spatial heterogeneity of antibody–drug conjugate targets in pancreatic ductal adenocarcinoma

**DOI:** 10.1002/2056-4538.70083

**Published:** 2026-03-16

**Authors:** Deema Sabtan, Marie‐Lisa Eich, Florian Loch, Julen Karl Pérez Zuschneid, Markus Möbs, Judith Böhme, Frederick Klauschen, David Horst, Mihnea P Dragomir, Gabriel Dernbach, Simon Schallenberg

**Affiliations:** ^1^ Institute of Pathology Charite, Universitätsmedizin Berlin, Corporate Member of Freie Universität Berlin and Humboldt Universität zu Berlin Berlin Germany; ^2^ Department of Surgery Charité – Universitätsmedizin Berlin, Corporate Member of Freie Universität Berlin and Humboldt‐Universität zu Berlin Berlin Germany; ^3^ German Cancer Consortium (DKTK) German Cancer Research Center (DKFZ), Partner Site Berlin Heidelberg Germany; ^4^ Berlin Institute of Health Charite, Universitätsmedizin Berlin Berlin Germany; ^5^ Institute of Pathology Ludwig Maximilians University Munich Germany; ^6^ Cancer Consortium (DKTK) German Cancer Research Center (DKFZ), Munich Partner Site Heidelberg Germany; ^7^ BIFOLD‐Berlin Institute for the Foundations of Learning and Data Berlin Germany; ^8^ Aignostics GmbH Berlin Germany; ^9^ Present address: Department of Pathology, Faculty of Medicine King Abdulaziz University Rabigh Saudi Arabia

**Keywords:** pancreatic ductal adenocarcinoma, antibody–drug conjugates, targeted therapy, spatial heterogeneity, c‐MET, TROP‐2, NECTIN4

## Abstract

Pancreatic ductal adenocarcinoma (PDAC) is a lethal disease, with limited therapeutic options, few patients showing targetable molecular changes. New therapeutic strategies are necessary. Antibody–drug conjugates (ADCs) have emerged as alternative therapeutic strategies across various cancer types. Herein, we analyze the expression and spatial heterogeneity (six cores per patients) of three ADC targets (c‐MET, NECTIN4, and TROP‐2) in a cohort of 62 PDAC patients (1,116 tissue cores) and associate their levels with clinicopathological and genomic parameters, and the expression of immune checkpoints. c‐MET exhibited significantly higher expression at the tumor front versus tumor center, along with notable intratumoral heterogeneity. In contrast, NECTIN4 and TROP‐2 displayed homogeneous expression patterns, with NECTIN4 being absent in approximately two‐thirds of cases, while TROP‐2 showed consistently strong positivity across tumor regions (98% 3+). By simulating sampling sufficiency for reliable scoring, we observed that, for c‐MET, two tumor samples were sufficient to achieve a maximum score of 1+, while for higher scores (2+ and 3+), four samples were required. For NECTIN4, four samples were necessary to detect scores of 1+ and 2+. For TROP‐2, for a 3+ score, just two samples were sufficient to reach the maximum score. c‐MET or TROP‐2 expression scores were not associated with any clinicopathological parameters. In contrast, NECTIN4 expression showed an association with tumor grade. Correlations with immune checkpoints revealed that high TROP‐2 expression was inversely correlated with PD‐L1 expression. For all three markers no significant differences in expression were found between *SMAD4* wild‐type and *SMAD4*‐mutated tumors, nor between *TP53* wild‐type and *TP53*‐mutated tumors. Furthermore, analysis of lymph node and distant (liver and peritoneal) metastases revealed significantly higher c‐MET and NECTIN4 expression in the metastatic setting. In conclusion, TROP‐2 is highly expressed in most PDACs, independent of clinicopathological and genomic parameters, and inversely correlating with PD‐L1, making TROP‐2 an ideal ADC target.

## Introduction

Pancreatic cancer is among the 10 most lethal malignancies worldwide, accounting for approximately 467,000 cancer‐related deaths annually [1]. Pancreatic ductal adenocarcinoma (PDAC) represents over 90% of these cases and is marked by late‐stage diagnosis and rapid disease progression, leading to a five‐year survival rate below 10% [2, 3]. The current standard of care includes surgical resection followed by adjuvant combination chemotherapy, such as FOLFIRINOX (fluorouracil, leucovorin, irinotecan, and oxaliplatin) [4]. Despite this aggressive regimen, median overall survival remains limited to 10–20 months [3, 4, 5, 6]. Additionally, the role of radiotherapy in PDAC remains controversial and for the currently approved targeted therapies [*KRAS* ^G12C^, homologous recombination deficiency, mismatch repair deficiency, and rare fusions (NRG1, NTRK)], only very few patients can profit [7].

Consequently, there is an urgent need for reliable biomarkers to guide and expand targeted therapy. Antibody–drug conjugates (ADCs) have emerged as a promising therapeutic strategy across various cancer types [8, 9]. ADCs consist of a monoclonal antibody (mAb) specific to a tumor‐associated antigen, a cleavable linker, and a cytotoxic payload. Following antigen binding and internalization, the payload is released intracellularly, disrupting essential cellular processes and inducing apoptosis [10].

Among the promising ADC targets is c‐MET, a receptor tyrosine kinase activated by hepatocyte growth factor (HGF), which plays a role in cell proliferation, migration, survival, and angiogenesis [11]. Its downstream signaling includes key oncogenic pathways such as Ras/Raf/MEK/ERK. ADCs targeting c‐MET and downstream effectors like MEK1/2 are under clinical evaluation [12, 13].

NECTIN4, a cell adhesion molecule of the immunoglobulin superfamily, contributes to cell–cell communication and has gained therapeutic relevance through the approval of enfortumab vedotin (EV), a NECTIN4‐targeting ADC for urothelial carcinoma [14, 15].

TROP‐2, a transmembrane glycoprotein overexpressed in multiple epithelial cancers, including PDAC, is targeted by agents such as sacituzumab govitecan and datopotamab deruxtecan. These agents are already approved in indications like breast cancer, non‐small cell lung cancer, and urothelial carcinoma [16, 17, 18, 19, 20]. In PDAC, some studies showed that TROP‐2 overexpression correlates with poor prognosis, metastatic potential, and reduced progression‐free survival [5, 17].

In this study, we investigated the spatial expression patterns and intratumoral heterogeneity of c‐MET, NECTIN4, and TROP‐2 in a cohort of 62 patients with PDAC and in unmatched lymph node metastases and distant liver and peritoneal metastases. We further examined correlations with immune checkpoint expression, mutational profiles, and histopathological features. Additionally, we assessed the number of sampled tumor regions per case to obtain a reliable expression score.

## Materials and methods

### Clinical cohorts

This retrospective, single‐center study was approved by the institutional ethics committee of Charité – Universitätsmedizin Berlin (approval number EA2/031/21). A total of 62 patients with histologically confirmed PDAC who underwent oncologic pancreatic resection after evaluation of an interdisciplinary tumor board between September 2009 and September 2020 at the Department of Surgery, Campus Benjamin Franklin, were included. Informed consent was obtained from all participants. Tumor tissue was retrieved from formalin‐fixed, paraffin‐embedded (FFPE) archival material. The median follow‐up duration was 61 months (range: 36–118 months).

In order to test the heterogeneity in ADC target expression between primary tumors and metastases we further added two previously published cohorts [21, 22]. Details regarding tissue microarray (TMA) construction can be found in the initial publications [21, 22, 23]. Altogether, these two cohorts contained: 21 PDAC liver metastases, 7 PDAC peritoneal carcinosis, 1 PDAC lung metastasis, and two lymph node metastases.

Finally, to explore the ADC target expression in lymph node metastases, we have included 19 additional samples for which the lymph nodes were analyzed.

### Tissue‐microarrays and immunohistochemistry

As described previously [24], FFPE tumor blocks were obtained from the archive of the Institute of Pathology, Charité – Universitätsmedizin Berlin. Two experienced pathologists with 5 and 10 years' experience in gastrointestinal pathology reviewed all cases to confirm tumor subtype, histological grade, and pathological stage, including assessment of vascular, lymphatic, and perineural invasion, according to the 8th edition of the TNM classification (AJCC). Tumor front (TF) and tumor center (TC) regions were defined and annotated by the pathologists on H&E‐stained whole‐slide sections prior to TMA construction (example images are shown in supplementary material. Figure S1). The TF was defined as the invasive tumor margin where tumor cells infiltrate adjacent pancreatic parenchyma, the duodenal wall, or peripancreatic adipose tissue, whereas the TC comprised central tumor areas without direct contact with surrounding non‐neoplastic tissue. For TMA construction, triplicates of 2‐mm tissue cores were sampled from both the TF and TC and embedded into recipient paraffin blocks. Lymphoid tissue was incorporated as an internal control.

An additional TMA was constructed, including unmatched lymph node metastases from 19 patients. For each sample, two 2‐mm tissue cores were sampled.

For immunohistochemical analysis, TMA blocks were sectioned at a thickness of 2 μm. Sections were pretreated with either CC1 mild buffer (Ventana Medical Systems, Tucson, AZ, USA) for 30 min at 100 °C or with protease 1 for 8 min. Antibody staining was performed using anti‐c‐MET (SP44, Roche/Ventana, ready‐to‐use), anti‐NECTIN4 (EPR15613‐68, Abcam, 1:50), or anti‐TROP‐2 (EPR20043, Abcam, 1:1000) antibodies, incubated for 60 min at room temperature. Visualization was carried out using the avidin–biotin complex method with DAB as chromogen. All immunostaining procedures were performed on a BenchMark XT immunostainer (Ventana Medical Systems). Counterstaining of nuclei was performed with hematoxylin and bluing reagent (Ventana Medical Systems) for 12 min. Stained sections were evaluated using Olympus BX50 and BX46 microscopes (Olympus Europe, Hamburg, Germany). As positive controls for NECTIN4 and TROP‐2, we used a previously published urothelial carcinoma tissue microarray containing the full spectrum of expression scores [25]. For c‐MET, non‐small cell lung cancer samples with known c‐MET expression were included as internal controls, as c‐MET immunohistochemistry is routinely performed and well established in our laboratory [26]. Immune checkpoint markers included IDO, PD‐L1, LAG‐3, TIM‐3, VISTA, and B7‐H4. Parts of the immune checkpoint markers results have been reported previously [24].

### Immunohistochemistry scoring

A single pathologist (DS) performed the scoring of all immunohistochemically stained tissue to ensure consistency throughout the study. Ambiguous cases were reviewed jointly with a second experienced pathologist (MPD) to achieve diagnostic consensus. Expression of c‐MET, NECTIN4, and TROP‐2 was assessed using the histochemical scoring system (*H*‐score), as previously described [25, 27, 28, 29]. Based on *H*‐score values, tissue cores were categorized as negative (*H*‐score 0–14), weakly positive/score 1 (*H*‐score 15–99), moderately positive/score 2 (*H*‐score 100–199), or strongly positive/score 3 (*H*‐score 200–300).

### Mutational analysis (next‐generation sequencing)

For all samples where material was available, tumor‐rich areas were marked on slides from a separate FFPE tissue block that had not been used for TMA construction using a light microscope (Olympus, BX46) by one of the study pathologists (MPD). Five to twenty 5 μm thick serial slides were microdissected for DNA extraction. DNA extraction was performed semi‐automated according to the manufacturer's instructions (Maxwell RSC FFPE Plus DNA Purification Kit, Custom, Promega, Madison, WI, USA). DNA quantification was performed using Qubit HS DNA assay (Thermo Fisher Scientific, Waltham, MA, USA).

For DNA sequencing, we generated the libraries using AmpliSeq for Illumina Cancer Hotspot Panel v2 (Illumina, San Diego, CA, USA) following the manufacturer's protocol. A total of 80 ng of genomic DNA from each sample was used as input. The sequencing panel covers the hotspot mutations of 50 genes with established pathogenic role in cancer: *ABL1*, *EGFR*, *GNAS*, *KRAS*, *PTPN11*, *AKT1*, *ERBB2*, *GNAQ*, *MET*, *RB1*, *ALK*, *ERBB4*, *HNF1A*, *MLH1*, *RET*, *APC*, *EZH2*, *HRAS*, *MPL*, *SMAD4*, *ATM*, *FBXW7*, *IDH1*, *NOTCH1*, *SMARCB1*, *BRAF*, *FGFR1*, *JAK2*, *NPM1*, *SMO*, *CDH1*, *FGFR2*, *JAK3*, *NRAS*, *SRC*, *CDKN2A*, *FGFR3*, *IDH2*, *PDGFRA*, *STK11*, *CSF1R*, *FLT3*, *KDR*, *PIK3CA*, *TP53*, *CTNNB1*, *GNA11*, *KIT*, *PTEN*, and *VHL*. The regions of interest were amplified by PCR (Biometra TOne, Analytik Jena, Jena, Germany) following the manufacturer's instructions. Next‐generation sequencing was performed using an iSeq 100 Sequencing System (Illumina).

For mutation calling we used the SEQUENCE Pilot Software version 5.4.0 (JSI Medical Systems GmbH, Ettenheim, Germany). In brief, sequencing reads were aligned to the human reference genome (hg19), and variant calling was performed using the software's integrated pipelines. An allele frequency greater than 5% was used as a threshold for selecting relevant mutations. Only mutations classified as pathogenic or potentially pathogenic based on established databases were selected for further analysis.

### Statistical analysis

Analyses were carried out in Python 3.11.6 using pandas 2.2.2, numpy 1.26.4, scipy 1.13.0, matplotlib 3.9.0, seaborn 0.13.2, lifelines 2.2.2, and scikit‐learn 1.5.0. Raw *H*‐scores for c‐MET, NECTIN4, and TROP‐2 (range 0–300) were converted to four immunohistochemical categories – 0 (0–14), 1 (15–99), 2 (100–199), and 3 (200–300). In a previous publication, IDO, PD‐L1, VISTA, TIM‐3, LAG‐3, and B7‐H4 were binned at 0–1%, 1–10%, 10–50%, and >50% positive cells and likewise encoded 0–3 [24]. For descriptive purposes, each patient was summarized either by the highest score across all tissue cores or by separate values for TC and TF.

Clustered heat‐maps of score distributions were generated with hierarchical clustering (Euclidean distance, complete linkage) of the percentage of cores scoring 0–3 for each marker. Sampling sufficiency was assessed using an urn‐experiment simulation to model sparse tissue sampling in the presence of intratumoral heterogeneity. For each patient, all available TMA cores (three from the TF and three from the TC, resulting in six cores per patient) were treated as the sampling pool. Tissue cores were repeatedly drawn without replacement, and after each draw it was evaluated whether the true patient‐level maximum expression score had been observed. This procedure was repeated 1,000 times per patient, and the average number of tissue cores required to capture the maximum expression score was calculated. To visualize inter‐patient variability, patients were subsequently ranked from low to high sampling requirement based on the average number of draws needed to observe the maximum score. This simulation framework was designed to quantify how many biopsy samples are required to reliably detect high target expression under conditions of spatial heterogeneity and has been described in detail in previous publications [25, 30]. Spatial heterogeneity was evaluated with a permutation test in which TF and TC labels were randomly reassigned 1,000 times; two‐sided *p* values equal the fraction of permutations whose mean difference matched or exceeded the observed mean difference. Permutation testing was chosen because protein expression data are frequently non‐normally distributed and may exhibit skewness or outliers; the median was therefore used as a robust measure of central tendency, as previously described [25]. The same approach was applied to compare primary tumors with unmatched metastases.

Overall survival (OS) – time from pancreatic resection to death or last follow‐up – served as the end‐point; patients alive at last contact were censored. Kaplan–Meier curves were compared with the log‐rank test, and Cox proportional‐hazards models assessed whether relative overexpression at the TF of each marker predicted OS after adjustment for log age at diagnosis, UICC stage, and resection margin status. Resection margin status was defined as margin status at the primary resection (R0 versus R1), with R0 indicating microscopically negative margins and R1 indicating microscopic residual tumor at the resection margin. Clinicopathological associations were explored in contingency tables, visualized as stacked bar plots, and tested with the *χ* ^2^ test. The full analysis pipeline (Jupyter notebooks and plotting scripts) will be made publicly available on GitHub upon publication.

## Results

### Clinicopathological parameters and ADC target expression

**Figure 1:**
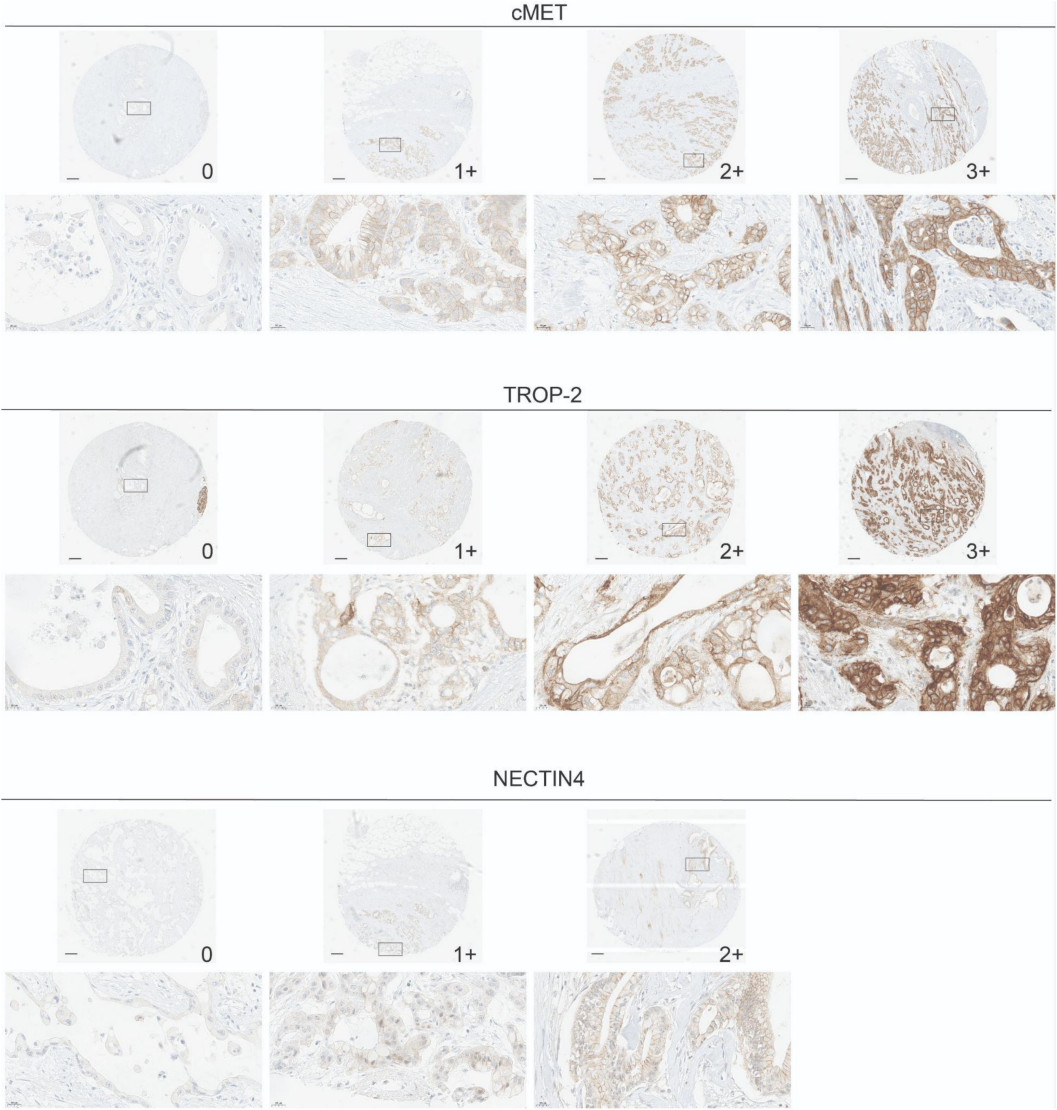
Staining patterns. Representative images of staining intensities for cMET (top), TROP‐2 (center), and NECTIN4 (bottom).

The study cohort consisted of 62 patients with PDAC; 58.1% male (*n* = 36) and 41.9% female (*n* = 26) (Table 1). The median age at diagnosis was 72 years (interquartile range: 67–76). All but one patient underwent upfront surgical resection; one patient received neoadjuvant chemotherapy prior to resection. Tumor stage distribution was as follows: pT1 in 3.2% (*n* = 2), pT2 in 25.8% (*n* = 16), and pT3 in 71.0% (*n* = 44). Lymph node metastases were present in 85.5% of cases (*n* = 53). Following the screening of a total of 1,116 tissue cores, 352/372 were evaluable for c‐MET, 357/372 for NECTIN4, and 360/372 for TROP‐2 analysis. A representative gallery of staining intensities for each marker is shown in Figure 1.

**Table 1:** Clinical and pathological characteristics of the cohort

Characteristics	Total
Age (years), median (interquartile range)	72 (67–76)
Gender	
Female	26
Male	36
T stage	
pT1	2
pT2	16
pT3	44
N stage	
N0	9
N1	53
Grade	
G1	1
G2	36
G3	25
Neoadjuvant chemotherapy	
Yes	1
No	61

### Spatial analysis of ADC targets in PDAC

**Figure 2:**
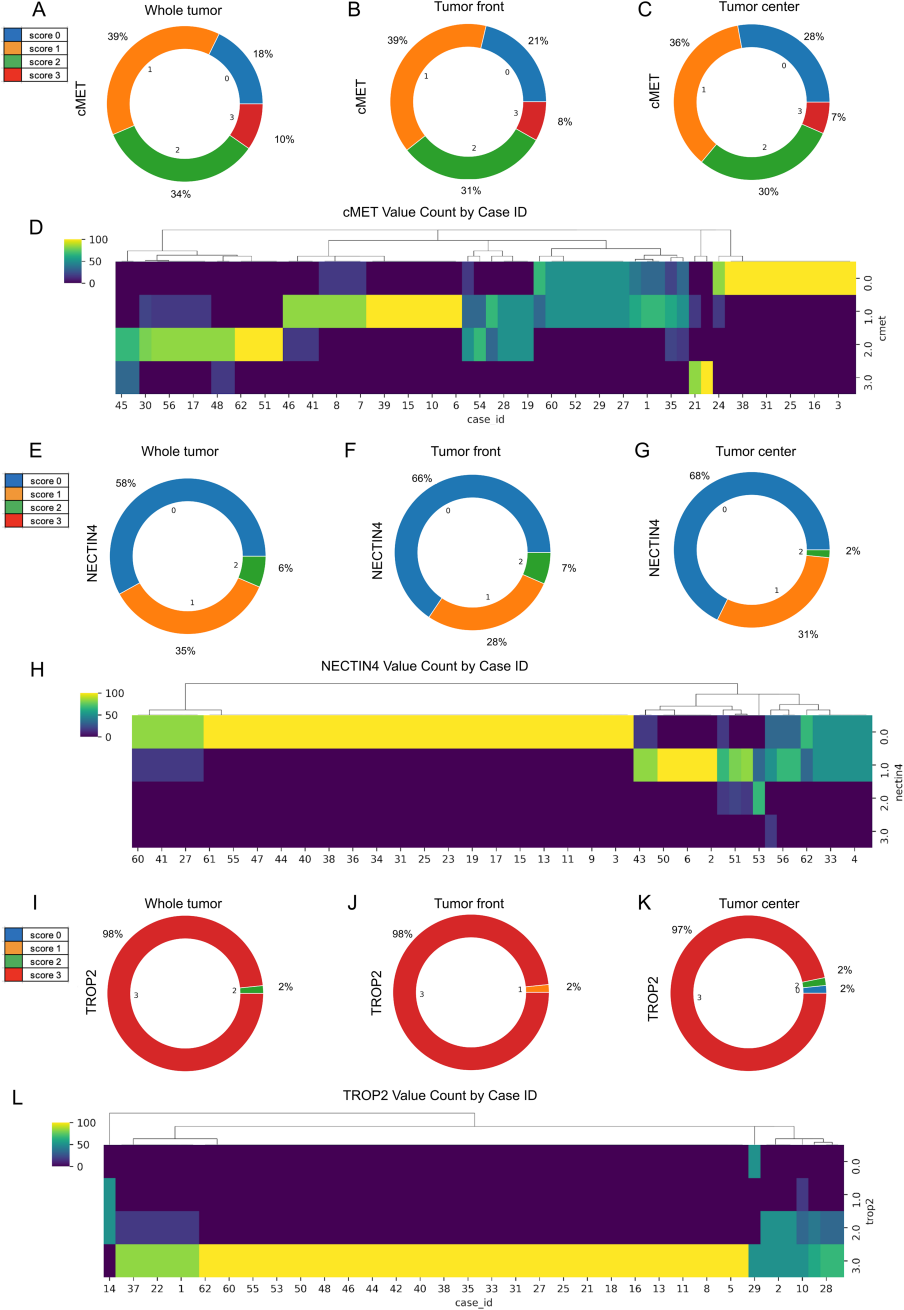
Heterogeneity assessment of c‐MET, NECTIN4, and TROP‐2. (A) Distribution of c‐MET immunohistochemistry (IHC) scores in the whole tumor, (B) in TF samples, and (C) TC samples. (D) Hierarchical clustering analysis with regard to IHC scoring for c‐MET. The color scale represents the proportion of tissue cores per patient assigned to each IHC score (0–3). Each patient is represented by six TMA cores; thus, 100% corresponds to all six cores and 50% to three of six cores showing the respective score. Yellow indicates the maximum proportion within a given score. (E) Distribution of NECTIN4 immunohistochemistry (IHC) scores in the whole tumor, (F) in TF samples, and (G) TC samples. (H) Hierarchical clustering analysis with regard to IHC scoring for NECTIN4. The color scale represents the proportion of tissue cores per patient assigned to each IHC score (0–3). Each patient is represented by six TMA cores; thus, 100% corresponds to all six cores and 50% to three of six cores showing the respective score. Yellow indicates the maximum proportion within a given score. (I) Distribution of TROP‐2 immunohistochemistry (IHC) scores in the whole tumor, (J) in TF samples, and (K) TC samples. (L) Hierarchical clustering analysis with regard to IHC scoring for TROP‐2. The color scale represents the proportion of tissue cores per patient assigned to each IHC score (0–3). Each patient is represented by six TMA cores; thus, 100% corresponds to all six cores and 50% to three of six cores showing the respective score. Yellow indicates the maximum proportion within a given score.

Analysis of c‐MET expression across the tumor revealed that the majority of cases exhibited a score of 1+ (39%), followed by 2+ (34%), 0 (18%), and 3+ (10%) (Figure 2A). When comparing spatial expression between TC and TF, a shift toward stronger positivity at the TF was observed (Figure 2B, C). Specifically, the proportion of negative cases decreased from 28% in the TC to 21% at the TF. This shift was statistically significant based on permutation testing (*p* = 0.007) (supplementary material, Figure S2A, B). An example case demonstrating higher expression at TF compared with TC is shown in supplementary material, Figure S2D. Hierarchical clustering of repeated samples indicated substantial intratumoral heterogeneity, with the most frequent mixed pattern consisting of scores 0 and 1 (Figure 2D). Given the invasive nature of the TF, we tested whether TF‐enriched expression of ADC targets was associated with disease‐free survival (DFS) independent of overall expression level, as a potential invasion‐associated spatial biomarker. However, relative overexpression of c‐MET at the TF compared to the TC was not significantly associated with DFS in multivariate Cox regression adjusted for UICC stage, patient age, and resection status (supplementary material, Figure S2C).

In contrast, NECTIN4 expression was negative in 58% of cases, with 35% showing a score of 1+ and only 6% a score of 2+ (Figure 2E). No significant spatial differences in expression between TC and TF were detected (*p* = 0.722; Figure 2F, G and supplementary material, Figure S2E, F). Sampling heterogeneity analysis showed limited variation, with most discordant cases fluctuating between scores 0 and 1+ (Figure 2H). Similar to c‐MET, spatial overexpression of NECTIN4 at the TF did not correlate with DFS in multivariate analysis (supplementary material, Figure S2G).

TROP‐2 was uniformly and strongly expressed, with 98% of cases showing a score of 3+ (Figure 2I). No spatial variation between TC and TF was observed (*p* = 0.461; Figures 2J, K and supplementary material, Figure S2H, I). Heterogeneity analysis confirmed a largely homogeneous expression profile, with only a few cases demonstrating variation between scores 2+ and 3+ (Figure 2L). Again, no significant association between spatial TROP‐2 expression and DFS was observed (supplementary material, Figure S2J).

In summary, c‐MET exhibited significantly higher expression at the TF along with notable intratumoral heterogeneity. In contrast, NECTIN4 and TROP‐2 displayed more homogeneous expression patterns, with NECTIN4 being absent in approximately two‐thirds of cases, while TROP‐2 showed consistently strong positivity across tumor regions.

### Association of ADC target expression and clinicopathological parameters

Different c‐MET expression scores were not significantly associated with any of the examined clinicopathological parameters, including sex, histological grade, lymphatic invasion, vascular invasion, perineural invasion, resection status, tumor stage, nodal stage, or UICC8 stage (supplementary material, Figure S3). In contrast, NECTIN4 expression showed a significant association with tumor grade; however, this association was driven by small subgroup sizes. NECTIN4‐negative tumors were more frequently grade 2 and less frequently grade 3, while the grade 1 tumor in the cohort showed higher NECTIN4 expression scores. Given the limited number of cases in individual grade categories, this finding should be interpreted cautiously and considered exploratory (supplementary material, Figure S4). However, no further clinicopathological variables were significantly associated with NECTIN4 expression (supplementary material, Figure S4). Similarly, no significant associations were observed between TROP‐2 expression scores and any of the assessed clinicopathological parameters (supplementary material, Figure S5).

### Association of ADC target expression in PDAC with and molecular alterations and immune checkpoint molecules

**Figure 3:**
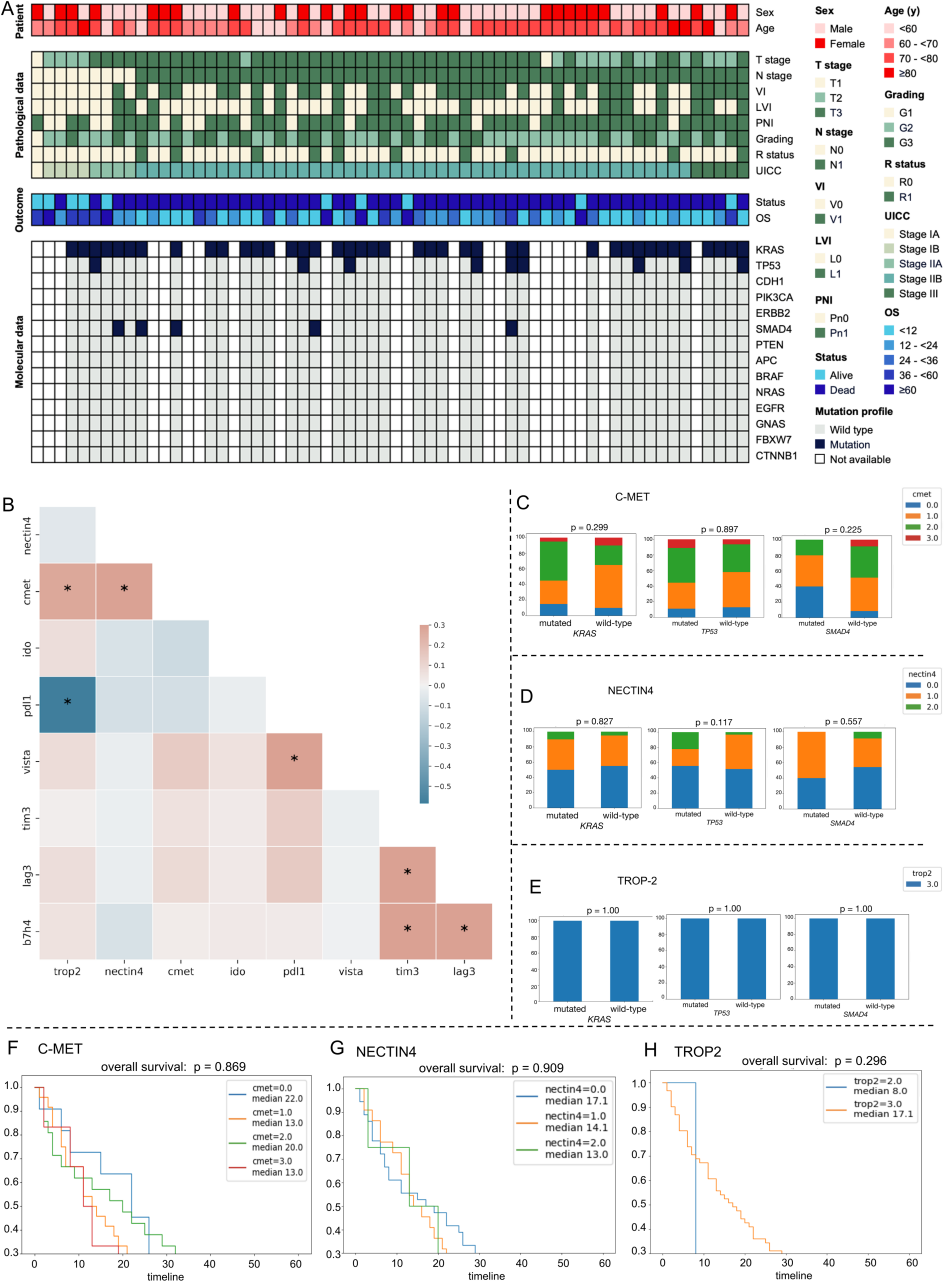
Association with clinicopathological data, immune checkpoint molecules, and molecular alterations. (A) Oncomap including clinicopathological data, ADC target expression, and molecular alterations. (B) Correlation matrix of ADC targets and immune checkpoint molecules. (C) Association of *KRAS* (left), *TP53* (center), and *SMAD4* mutations with c‐MET expression scores. (D) Association of *KRAS* (left), *TP53* (center), and *SMAD4* mutations with NECTIN4 expression scores. (E) Association of *KRAS* (left), *TP53* (center), and *SMAD4* mutations with TROP‐2 expression scores. Kaplan–Meier overall survival curves with corresponding risk tables for c‐MET (F), NECTIN4 (G), and TROP‐2 (H), stratified by immunohistochemical (IHC) scores (0–3). For NECTIN4, no samples exhibited a score of 3+, whereas for TROP‐2, no samples were scored 0 or 1+.

To give an overview of potential links between ADC target expression, molecular alterations, and immune checkpoint molecule expression, we generated an Oncomap (Figure 3A) that displays TN‐stage, tumor grading, clinical outcome, and sequencing data for each patient in the cohort. The most frequently mutated gene was *KRAS* (*n* = 40; 64.5%), followed by *TP53* (*n* = 9; 14.5%) and *SMAD4* (*n* = 4; 6.5%). Analysis of the immune checkpoint molecules revealed that high TROP‐2 expression was inversely correlated with PD‐L1 expression (*r* = −0.5). TROP‐2 expression also showed a moderate positive correlation with c‐MET expression (*r* = 0.3). Further correlations were observed between TIM‐3 and B7‐H4/LAG‐3 expression (*r* = 0.3 each), between PD‐L1 expression on tumor cells and VISTA (*r* = 0.3), and between LAG‐3 and B7‐H4 (*r* = 0.3) (Figure 3B). We next assessed ADC target expression in relation to *SMAD4* and *TP53* mutation status. For all three markers – c‐MET, NECTIN4, and TROP‐2 – no significant differences in expression were found between *SMAD4* wild‐type and *SMAD4*‐mutated tumors, nor between *TP53* wild‐type and *TP53*‐mutated tumors (Figure 3C–E). Finally, overall survival analyses were performed for all ADC targets using both individual expression scores (0–3) and a dichotomized expression approach (low: scores 0–1 versus high: scores 2–3). No significant differences in overall survival were observed for any of the ADC targets (Figure 3F–H and supplementary material, Figure S6A–C).

### Sampling accuracy of ADC targets in PDAC

**Figure 4:**
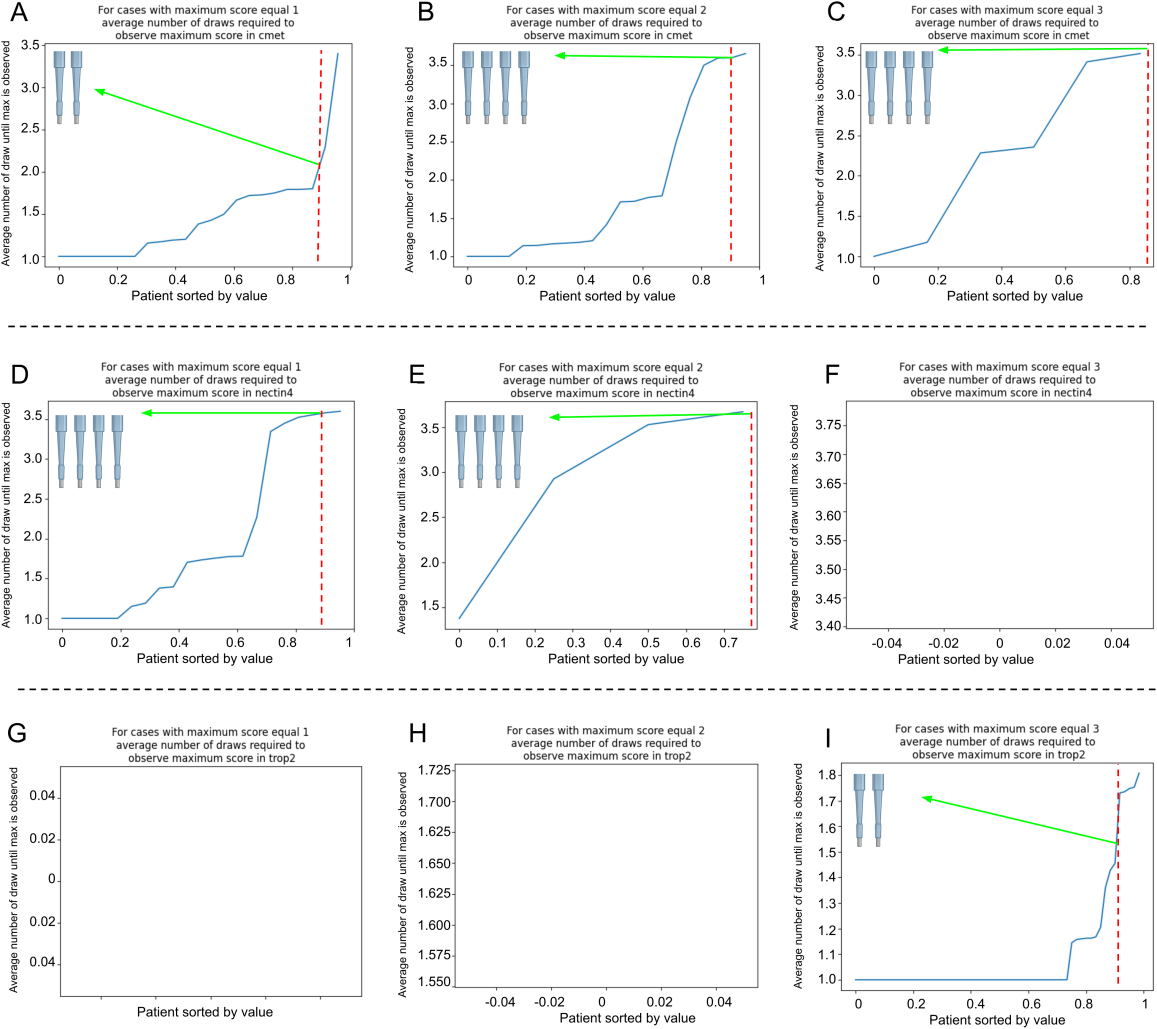
Sampling error: Urn problem experiment to detect sampling error across cases. The average number of draws representing the average number of biopsies to be scored are displayed on the *y*‐axis. Each marker was divided by immunohistochemistry score: (A) c‐MET score 1+, (B) c‐MET score 2+, (C) c‐MET score 3+, (D) NECTIN4 score 1+, (E) NECTIN4 score 2+, (F) NECTIN4 score 3+, (G) TROP‐2 score 1+, (H) TROP‐2 score 2+, and (H) TROP‐2 score 3+. c‐MET, hepatocyte growth factor receptor; NECTIN4, nectin cell adhesion molecule 4; TROP‐2, trophoblast cell surface antigen 2.

To assess sampling sufficiency for reliable scoring, we estimated how many tumor samples per patient were required to capture the true patient‐level maximum expression score using an urn‐experiment simulation designed to model sparse tissue sampling typically encountered in routine diagnostic biopsy settings in the presence of intratumoral heterogeneity. Each patient contributed six TMA cores (three from the TF and three from the TC), which served as the sampling pool for the simulation analysis. A sampling strategy was considered sufficient if the maximum score was correctly identified in at least 90% of patients. For c‐MET, two tumor samples were sufficient to achieve a maximum score of 1+ in over 90% of cases (Figure 4A). For higher scores (2+ and 3+), four samples were required to reliably reach the maximum (Figure 4B, C). For NECTIN4, four samples were necessary to detect scores of 1+ and 2+ in more than 90% of cases (Figure 4D, E). Cases with a score of 3+ could not be analyzed due to insufficient sample numbers (Figure 4F). Similarly, for TROP‐2, sample numbers were insufficient to analyze scores of 1+ and 2+ (Figure 4G, H). However, for the most frequent score of 3+, just two samples per patient were sufficient to reach the maximum in over 90% of cases (Figure 4I).

### ADC target expression in PDAC lymph node and distant metastases

**Figure 5:**
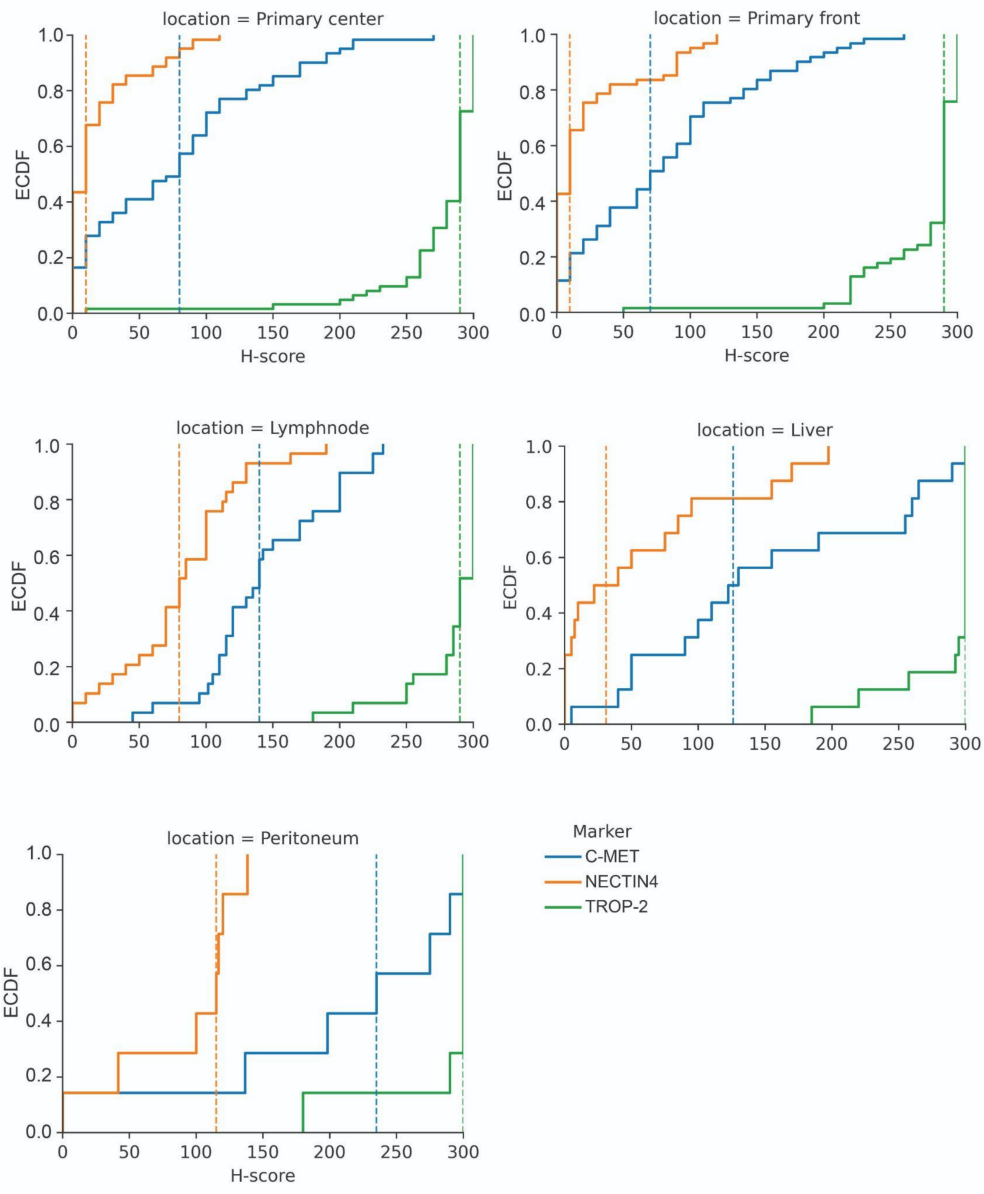
Distribution of ADC targets (c‐MET, NECTIN4, and TROP‐2) across PDAC primary center, PDAC primary front, PDAC lymph node metastases, PDAC liver metastases, and PDAC peritoneal metastases. The vertical lines represent the median expression for a given ADC target in a given localization. For statistical tests, see supplementary material, Table S1.

Finally, to capture the full spectrum of ADC target spatial heterogeneity, we also analyzed unmatched lymph node, liver, and peritoneal PDAC metastases and compared their protein expression with that of primary PDAC (TF and TC separately). Differences between anatomical locations were assessed using a permutation test on the median ADC target expression (Figure 5), chosen for robustness to non‐normal and skewed expression distributions.

For c‐MET we observed significantly higher values in lymph node metastases versus primary TF (*p* = 0.0006) or TC (*p* = 0.0018), in peritoneal metastases versus primary TF (*p* = 0.0008) or TC (*p* = 0.0009), in peritoneal metastases versus lymph node metastases (*p* = 0.0042), and in liver metastases versus primary TF (*p* = 0.0338) or TC (*p* = 0.0389; Figure 5 and supplementary material, Table S1). Overall, these analyses revealed higher levels of c‐MET in metastases versus primary tumors.

For NECTIN4 we noticed significantly higher levels in lymph node metastases versus primary TF (*p* = 0.0034) or TC (*p* = 0.0002), in peritoneal metastases versus primary TF (*p* = 0.0016) or TC (*p* = 0.0004), in liver metastases versus primary TF (*p* = 0.0288) or TC (*p* = 0.0149), and in lymph node metastases versus liver metastases (*p* = 0.0264; Figure 5 and supplementary material, Table S1). Hence, also for NECTIN4 we observed higher expression in the metastatic setting.

For TROP‐2 expression, we did not observe any significant differences between all the different spatial localizations (Figure 5 and supplementary material, Table S1).

## Discussion

ADCs have emerged as a promising therapeutic modality. Recent preclinical and early clinical studies have explored several targets for ADC therapy in pancreatic cancer. In the current study, we have investigated the spatial distribution of three ADC target molecules, c‐MET, NECTIN4, and TROP‐2 in a cohort of 62 patients with PDAC and correlated marker expression with clinicopathological parameters and the immune checkpoint molecule expression.

Overexpression of the tyrosine kinase receptor c‐MET has been associated with poor survival in patients with PDAC. However, the criteria for defining overexpression have varied across studies [31]. In our study, we show that the majority of tumors displayed a score of 1+. Regarding spatial distribution, higher c‐MET scores were observed at the TF compared to the TC, and tumors also demonstrated notable intratumoral heterogeneity. To the best of our knowledge, this is the first study to examine differential c‐MET expression between the TC and TF. A small study including 12 patients with PDAC also reported intratumoral heterogeneity in c‐MET expression [32]. However, these differences should be interpreted with caution, especially when the absolute differences are small (in our case, 7%). Accordingly, TF‐TC comparisons are presented as descriptive analyses that capture spatial expression gradients that may be relevant in a biopsy situation and whose biological significance requires further investigation. To accurately determine c‐MET status, a minimum of four biopsies was required for tumors with a c‐MET score of 2+ or 3+. Currently, no standardized recommendations exist regarding the number of biopsies needed for reliable marker expression scoring in PDAC. A study evaluating c‐MET expression in 131 PDAC patients reported a strong correlation between scores obtained from whole‐slide sections and TMA cores, but did not address the number of biopsies required for accurate assessment [33]. In contrast to the positive correlation reported between c‐MET and PD‐L1 expression in hepatocellular carcinoma cell lines [34], we did not observe any correlation of c‐MET expression with immune checkpoint molecule expression or sequencing data in our PDAC cohort.

In a previous study, NECTIN4 expression was observed in 71% of 164 examined PDACs [27]. In contrast, the majority of tumors in our cohort were negative for NECTIN4 (58%), with only a small proportion showing moderate (6%) staining, but in a metastatic setting we observed somewhat higher levels and closer to the already published literature, reaching 33% moderate staining. Defining NECTIN4‐high as ≥50% of tumor cells staining positive, Nishiwada *et al* reported poorer overall survival in patients with NECTIN4‐high tumors [35]. In our study, using a four‐tier IHC‐based scoring system, we did not observe any correlation between NECTIN4 expression and patient outcomes. Grade 1 tumors occurred only in cases with a NECTIN4 score of 2+, whereas tumors with scores of 0 or 1+ were exclusively grade 2 or 3. Similar to our findings with c‐MET, at least four biopsies were required to reliably determine NECTIN4 scores. No significant correlations were found between NECTIN4 expression and immune marker levels. Consistent with a pan‐cancer analysis of mRNA expression of potential ADC targets in the TCGA cohort [36], we observed a weak positive correlation between c‐MET and NECTIN4 expression.

TROP‐2 is overexpressed in many epithelial tumors [37]. Early studies of PDAC using a different antibody reported that TROP‐2 overexpression correlated with aggressive clinicopathological features, including higher tumor grade and lymph node metastases [17]. In contrast, and consistent with our findings, Mas *et al*, using the same antibody clone as our study, found no correlation between TROP‐2 expression and clinicopathological parameters. Furthermore, they observed weak staining equivalent to our TROP‐2 score of 1+ in only 2% of cases, with the remaining cases showing at least moderate positivity [38]. In our study, TROP‐2 expression was homogeneous, suggesting that an accurate TROP‐2 score can be obtained with only two biopsies. We next examined correlations with molecular sequencing data and did not observe significant differences in TROP‐2 expression among tumors harboring *KRAS*, *TP53*, or *SMAD4* mutations. By contrast, Mas *et al* reported higher mRNA levels of TROP‐2 in tumors with *KRAS* and *TP53* mutations [38]. When analyzing the tumor immune microenvironment, we observed a negative correlation between TROP‐2 expression and PD‐L1 levels. This aligns with previous findings showing that patients with TROP‐2–high non–small cell lung cancer were less responsive to anti–PD‐L1 therapies [39]. A similar trend was reported in breast cancer, where patients with TROP‐2–high tumors identified by sequencing showed reduced response to PD‐1 blockade [40].

The translation of ADC target expression into therapeutic response remains an area of active investigation, particularly in PDAC, where no ADCs are currently approved and predictive biomarkers are lacking. Our finding that TROP‐2 is homogeneously expressed across most primary PDACs and PDAC metastases supports its potential as a clinically viable ADC target with limited sampling bias. Notably, the observed inverse correlation between TROP‐2 and PD‐L1 expression may suggest particular relevance in immunologically ‘cold’ PDAC subtypes, which are typically resistant to immune checkpoint blockade [41]. We now emphasize this observation as a potential biomarker‐driven rationale for patient selection in future ADC‐based therapeutic strategies.

In contrast to reports describing a positive association between c‐MET and PD‐L1 expression in other malignancies, including hepatocellular carcinoma [34], we did not observe any correlation between c‐MET expression and immune checkpoint molecules in our PDAC cohort. This lack of association suggests that, in PDAC, c‐MET expression may not be directly linked to immune evasion mechanisms. Importantly, this implies that c‐MET–targeted ADCs may retain therapeutic relevance in immunologically neutral tumors, where checkpoint inhibitors have shown limited efficacy.

Finally, while enfortumab vedotin, a NECTIN4‐targeted ADC, is approved for urothelial carcinoma without mandatory pre‐treatment biomarker assessment [42, 43], emerging evidence indicates that NECTIN4 expression levels may still influence therapeutic response, particularly in the context of resistance [44]. Although these observations derive from non‐PDAC settings, they underscore the broader principle that target expression may serve as a clinically relevant stratification factor even when not required for trial eligibility.

This study is not without limitations. While the use of TMAs is well established for analyzing large patient cohorts, they may not fully capture intratumoral heterogeneity (ITH) due to the limited sampling area. Future studies should incorporate whole‐tumor analyses to provide a more comprehensive understanding of marker distribution. Secondly, our analysis was based on a single‐center cohort of 62 patients, which may limit the generalizability of our findings. Larger, multi‐center studies are needed to validate these results and improve statistical power. Moreover, due to the modest cohort size and sparsely populated ordinal categories, clinicopathological associations were analyzed in an exploratory manner, and multivariable regression analyses were not feasible. Thirdly, as no ADCs are currently approved for the treatment of pancreatic cancer, we were unable to assess any associations with treatment response. Finally, our molecular characterization was limited to hotspot mutations in a targeted 50‐gene panel, and we did not assess genomic, transcriptomic, or copy number alterations of ADC targets. While such analyses may provide complementary biological insights, the present study deliberately focused on protein expression, which represents the clinically relevant level for ADC binding and activity [45]. Future studies integrating genomic, transcriptomic, and proteomic data will be required to more comprehensively characterize ADC target heterogeneity in PDAC.

Our findings underscore the importance of multiple sampling to accurately assess intratumoral heterogeneity, particularly for c‐MET and NECTIN4 expression in PDAC. In contrast, TROP‐2 expression was homogeneous and diffusely high, suggesting that limited sampling may still yield representative results. Its inverse correlation with PD‐L1 expression provides a potential rationale for the use of sacituzumab govitecan in treating ‘immune‐cold’ tumors.

## Author contributions statement

DS, M‐LE, FL, MPD, GD and SS designed the study, selected the cases and collected and assembled data. DS, M‐LE, JKPZ, MPD, GD and SS performed the analysis. FL, MM, JB, FK and DH provided data support. DS, M‐LE, MPD, GD and SS drafted the manuscript. JKPZ, FL, MM, JB, FK and DH reviewed the manuscript and provided feedback with edits. All authors read and approved the final paper.

## Supporting information


**Figure S1.** Representative H&E‐stained whole‐slide section illustrating the definition of tumor center and tumor front
**Figure S2.** Spatial heterogeneity and survival impact of c‐MET, NECTIN4, and TROP‐2 expression
**Figure S3.** Association of clinical parameters and c‐MET expression
**Figure S4.** Association of clinical parameters and NECTIN4 expression
**Figure S5.** Association of clinical parameters and TROP‐2 expression
**Figure S6.** Kaplan–Meier overall survival curves with corresponding risk tables for c‐MET, NECTIN4, and TROP‐2
**Table S1.** Statistical comparison of ADC target distribution

## Data Availability

The data that support the findings of this study are available from the corresponding authors upon reasonable request.
