# Supplementary material for: Spatial heterogeneity of antibody–drug conjugate targets in pancreatic ductal adenocarcinoma
Source: J Pathol Clin Res. 2026 Mar 16;12(2):e70083. doi: 10.1002/2056-4538.70083 (PMC13093404; doi:10.1002/2056-4538.70083)
Supplement: Supplementary file 1 — Figure S1. Representative H&E‐stained whole‐slide section illustrating the definition of tumor center and tumor front Figure S2. Spatial heterogeneity and survival impact of c‐MET, NECTIN4, and TROP‐2 expression Figure S3. Association of clinical parameters and c‐MET expression Figure S4. Association of clinical parameters and NECTIN4 expression Figure S5. Association of clinical parameters and TROP‐2 expression Figure S6. Kaplan–Meier overall survival curves with corresponding risk tables for c‐MET, NECTIN4, and TROP‐2 Table S1. Statistical comparison of ADC target distribution [file CJP2-12-e70083-s001.pdf]

Spatial heterogeneity of antibody–drug conjugate targets in pancreatic ductal adenocarcinoma

Deema Sabtan, Marie-Lisa Eich, Florian Loch *et al*. *J Pathol Clin Res*
https://doi.org/10.1002/2056-4538.70083

Supplementary Figures S1–S6

Supplementary Table S1

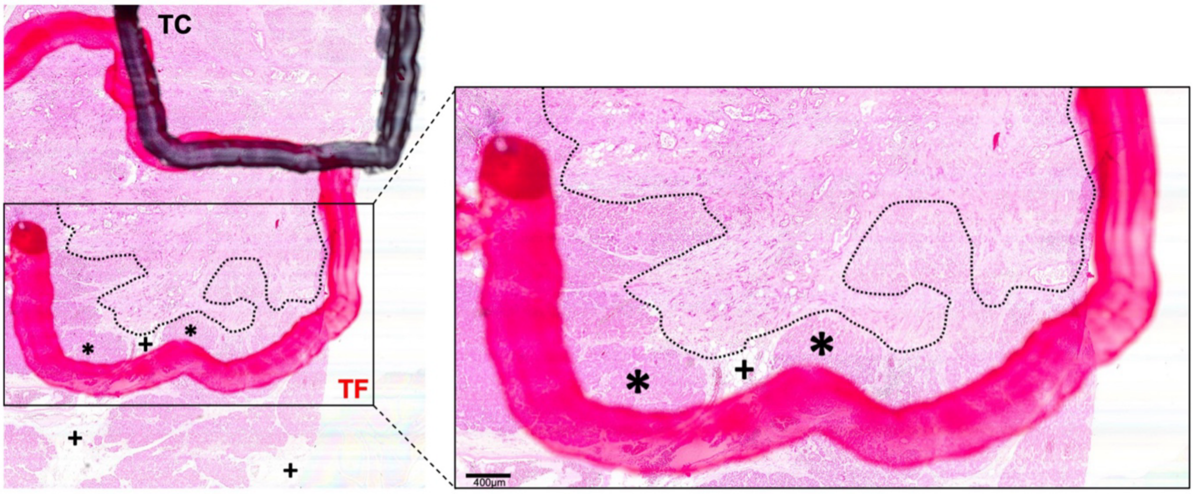

Figure S1. Representative H&E-stained whole-slide section illustrating the definition of tumor center (TC) and tumor front (TF). On the left, the overview shows TC annotated in black by the pathologist (upper panel) and TF outlined in red (lower panel). The dashed line indicates the invasive tumor front. Asterisks mark adjacent acinar pancreatic parenchyma, and plus signs indicate peripancreatic adipose tissue. On the right, a higher magnification view highlights the invasive tumor front.

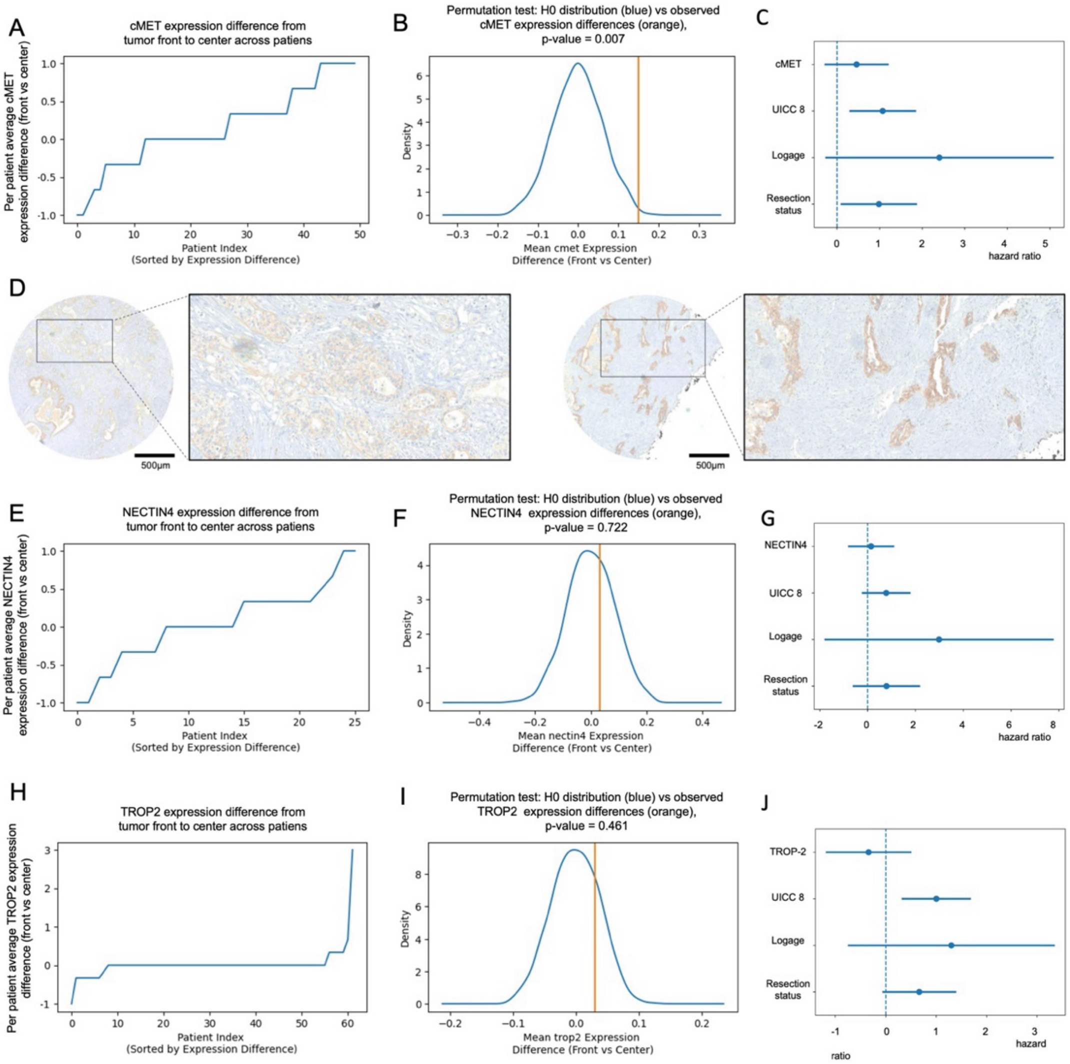

Figure S2. Spatial heterogeneity and survival impact of c-MET, NECTIN4, and TROP-2 expression. (A) Within-patient difference in expression from front to center region, with permutation test in (B) to assess statistical significance of the difference in c-MET expression in the whole cohort. (C) Cox regression analysis for relative overexpression of c-MET at the TF compared to the TC and disease-free survival (DFS) time including Union Internationale Contre Le Cancer (eighth edition) stage, log patient age (years) at diagnosis, and resection status. Scoring: c-MET: 0 = score 0, 1 = score 1, 2 = score 2, and 3 = score 3. Log-age, log patient age at diagnosis. (D) Representative case illustrating spatial heterogeneity of c-MET expression. On the left, a tumor center (TC) tissue core and corresponding higher magnification image show weak c-MET expression (score 1). On the right, a tumor front (TF) tissue core and corresponding higher magnification image demonstrate moderate c-MET expression (score 2). (E) Within-patient difference in expression from front to center region, with permutation test in (F) to assess statistical significance of the difference in NECTIN4 expression in the whole cohort. (G) Cox regression analysis for relative overexpression of NECTIN4 at the TF compared to the TC and disease-free survival (DFS) time including Union Internationale Contre Le Cancer (eighth edition) stage, log patient age (years) at diagnosis, and resection status. Scoring: NECTIN4: 0 = score 0, 1 = score 1, 2 = score 2, and 3 = score 3. Log-age, log patient age at diagnosis. (H) Within-patient difference in expression from front to center region, with permutation test in (I) to assess statistical significance of the difference in TROP-2 expression in the whole cohort. (J) Cox regression analysis for relative overexpression of TROP-2 at the TF compared to the TC and disease-free survival (DFS) time including Union Internationale Contre Le Cancer (eighth edition) stage, log patient age (years) at diagnosis, and resection status. Scoring: TROP-2: 0 = score 0, 1 = score 1, 2 = score 2, and 3 = score 3. Log-age, log patient age at diagnosis.

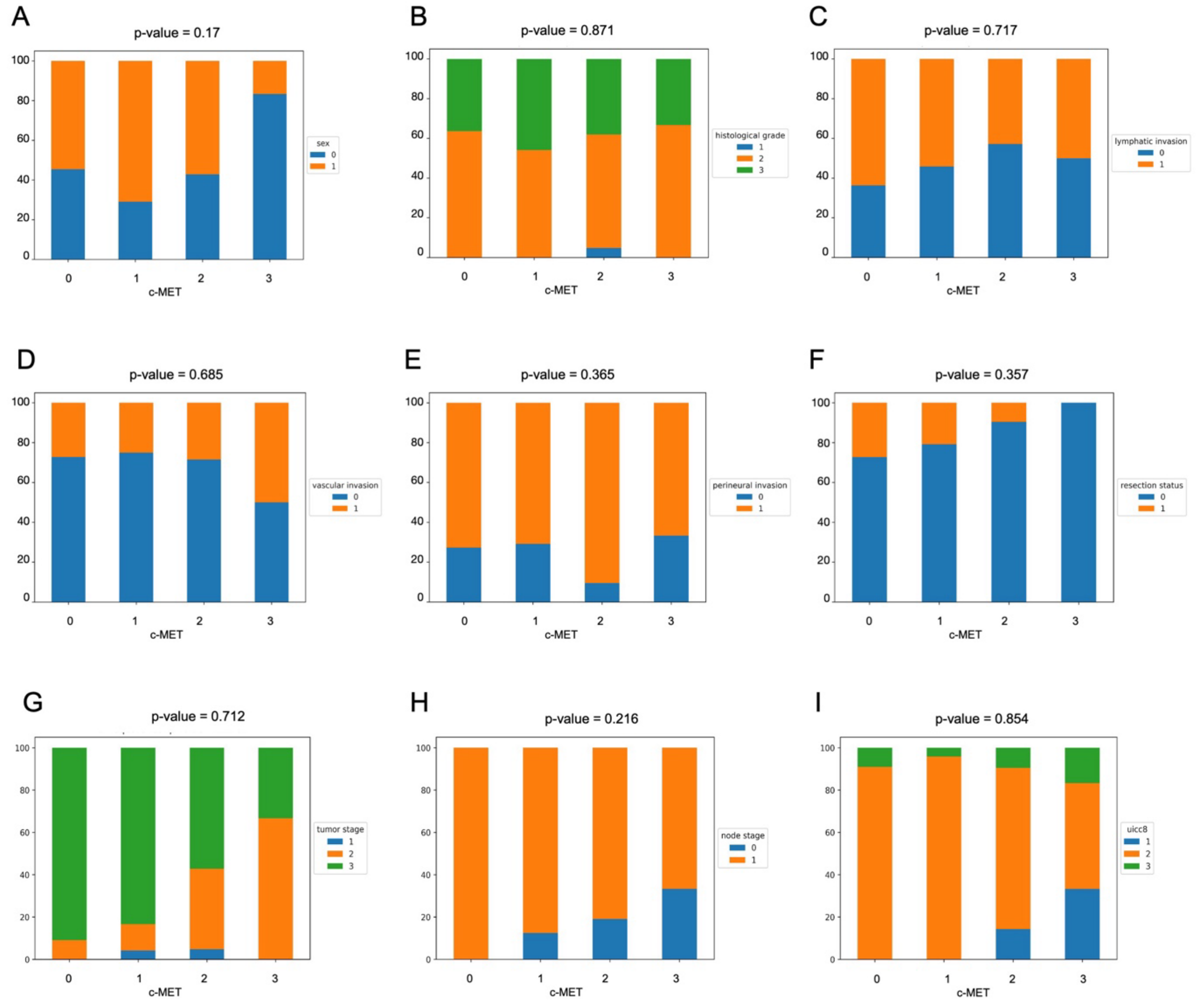

Figure S3. Association of clinical parameters and c-MET expression: (A) sex, (B) grade, (C) lymphatic invasion, (D) vascular invasion, (E) perineural invasion, (F) resection status, (G) tumor stage, (H) node stage, and (I) UICC8 stage.

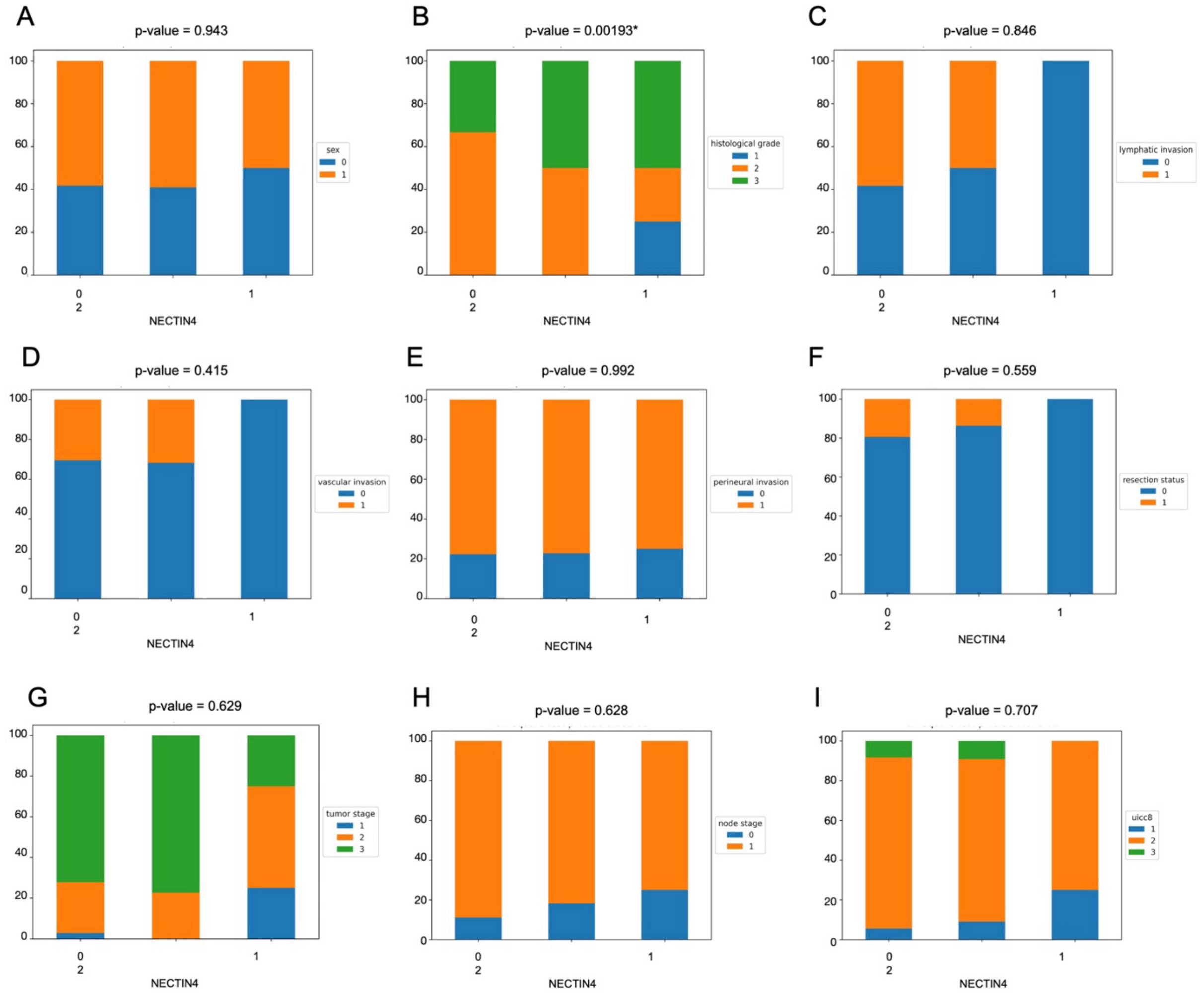

Figure S4. Association of clinical parameters and NECTIN4 expression: (A) sex, (B) grade, (C) lymphatic invasion, (D) vascular invasion, (E) perineural invasion, (F) resection status, (G) tumor stage, (H) node stage, and (I) UICC8 stage.

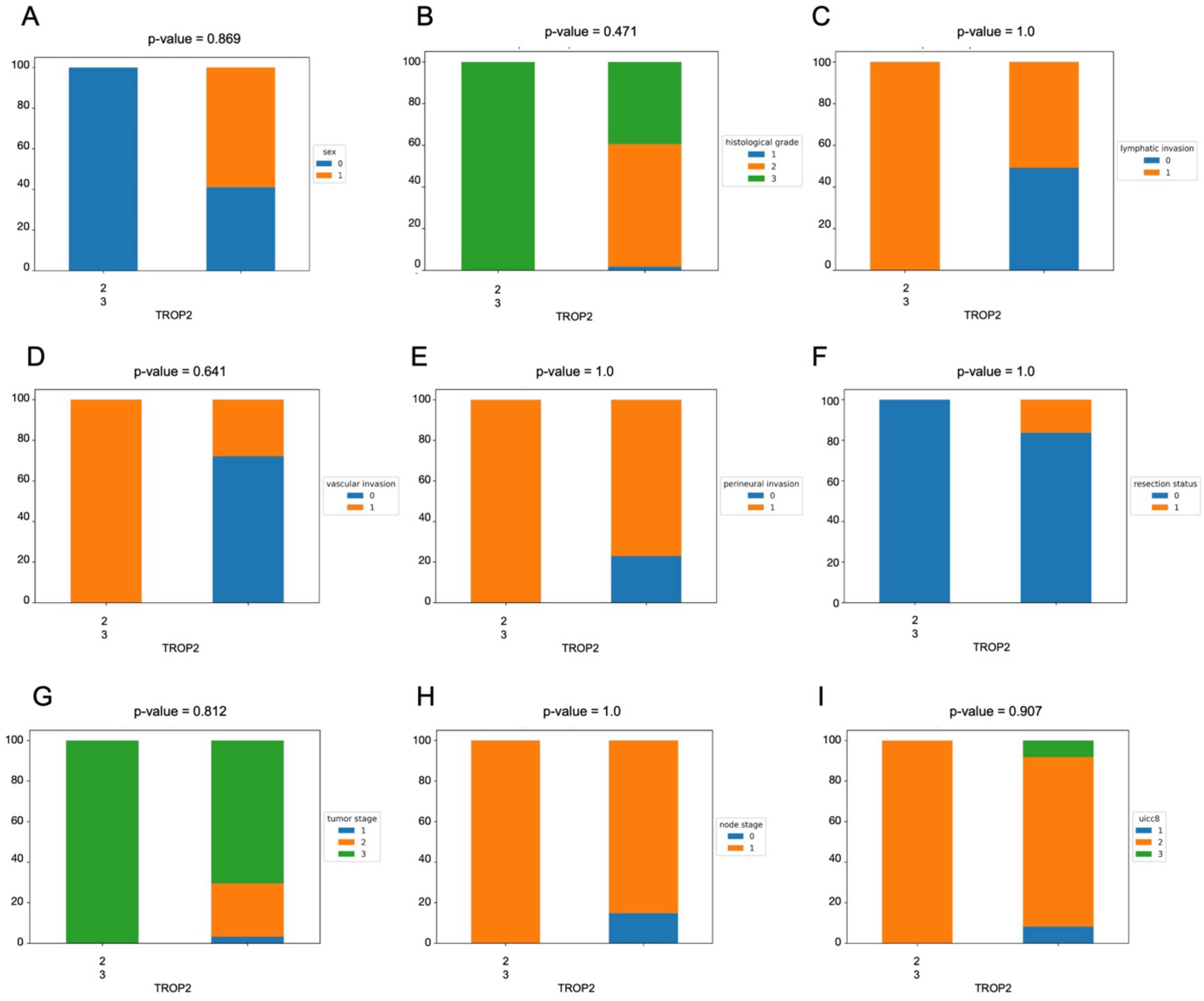

Figure S5. Association of clinical parameters and TROP-2 expression: (A) sex, (B) grade, (C) lymphatic invasion, (D) vascular invasion, (E) perineural invasion, (F) resection status, (G) tumor stage, (H) node stage, and (I) UICC8 stage.

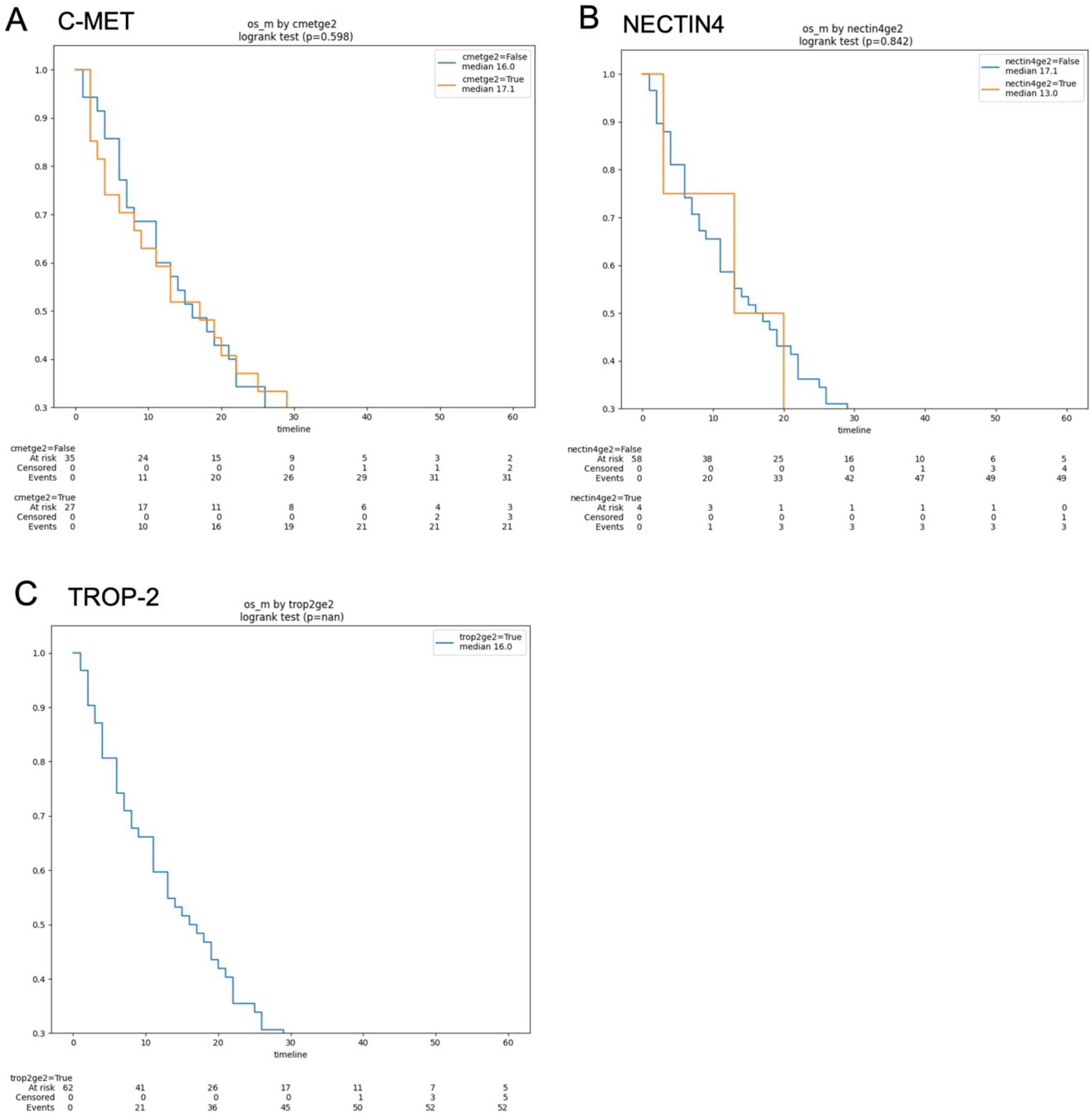

Figure S6. Kaplan–Meier overall survival curves with corresponding risk tables for (A) c-MET, (B) NECTIN4, and (C) TROP-2, stratified by a dichotomized expression approach (low: scores 0–1 vs high: scores 2–3). For TROP-2, no samples exhibited a low expression (scores 0-1).

Table S1. Statistical comparison of ADC targets distribution (c-MET, NECTIN4 and TROP-2) across PDAC primary center, PDAC primary front, PDAC lymph node metastases, PDAC liver metastases, and PDAC peritoneal metastases.

val	pval	low	high	loc1	loc2	marker
70	0.0002	60	95	Lymph node	Primary center	NECTIN4
105	0.0004	31.6667	116.667	Peritoneum	Primary center	NECTIN4
70	0.0006	30	100	Lymph node	Primary front	c-MET
165	0.0008	46.6667	230	Peritoneum	Primary front	c-MET
155	0.0009	56.6667	235	Peritoneum	Primary center	c-MET
105	0.0016	31.6667	116.667	Peritoneum	Primary front	NECTIN4
60	0.0018	35	110	Lymph node	Primary center	c-MET
70	0.0034	60	90	Lymph node	Primary front	NECTIN4
-95	0.0042	-160	5.83333	Lymph node	Peritoneum	c-MET
21	0.0149	-5	75	Liver	Primary center	NECTIN4
-49	0.0264	-91.25	5	Liver	Lymph node	NECTIN4
21	0.0288	-5	75	Liver	Primary front	NECTIN4
56.25	0.0338	0	175	Liver	Primary front	c-MET
46.25	0.0389	5	175	Liver	Primary center	c-MET
10	0.0612	5	10	Liver	Primary front	TROP-2
10	0.0624	2.5	15	Liver	Lymph node	TROP-2
-84	0.0849	-112.5	16	Liver	Peritoneum	NECTIN4
10	0.1092	5	20	Liver	Primary center	TROP-2
-35	0.1216	-50	58.3333	Lymph node	Peritoneum	NECTIN4
10	0.2046	0	10	Peritoneum	Primary front	TROP-2
10	0.2969	0	20	Peritoneum	Primary center	TROP-2
108.75	0.3429	-185	56.6667	Liver	Peritoneum	c-MET
-10	0.5101	-15	10	Lymph node	Peritoneum	TROP-2
-13.75	0.6407	-65	115	Liver	Lymph node	c-MET
0	1	-5	10	Lymph node	Primary front	TROP-2
0	1	-5	15	Lymph node	Primary center	TROP-2
0	1	-5	10	Liver	Peritoneum	TROP-2
